# An Appraisal of Lung Nodules Automatic Classification Algorithms for CT Images

**DOI:** 10.3390/s19010194

**Published:** 2019-01-07

**Authors:** Xinqi Wang, Keming Mao, Lizhe Wang, Peiyi Yang, Duo Lu, Ping He

**Affiliations:** 1 School of Software, Northeastern University, Shenyang 110004, China; wangxinqi7@163.com (X.W.); yangpeiyineu@163.com (P.Y.); lu.d.0@foxmail.com (D.L.); 2 Norman Bethune Health Science Center of Jilin University, No. 2699 Qianjin Street, Changchun 130012, China; wanglizhe2005@163.com; 3 School of Computer Science and Engineering, Northeastern University, Shenyang 110004, China; heping2598@163.com

**Keywords:** lung nodule classification, computer tomography, medical image analysis, pattern recognition

## Abstract

Lung cancer is one of the most deadly diseases around the world representing about 26% of all cancers in 2017. The five-year cure rate is only 18% despite great progress in recent diagnosis and treatment. Before diagnosis, lung nodule classification is a key step, especially since automatic classification can help clinicians by providing a valuable opinion. Modern computer vision and machine learning technologies allow very fast and reliable CT image classification. This research area has become very hot for its high efficiency and labor saving. The paper aims to draw a systematic review of the state of the art of automatic classification of lung nodules. This research paper covers published works selected from the Web of Science, IEEEXplore, and DBLP databases up to June 2018. Each paper is critically reviewed based on objective, methodology, research dataset, and performance evaluation. Mainstream algorithms are conveyed and generic structures are summarized. Our work reveals that lung nodule classification based on deep learning becomes dominant for its excellent performance. It is concluded that the consistency of the research objective and integration of data deserves more attention. Moreover, collaborative works among developers, clinicians, and other parties should be strengthened.

## 1. Introduction

**Figure 1:**
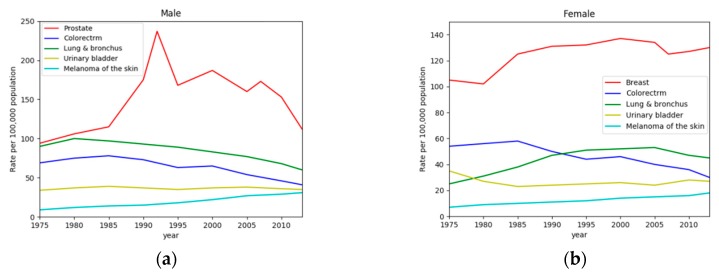
Trend of incidence rates for several cancers in the United States from 1975 to 2013. (**a**) and (**b**) present the trend on males and females, respectively.

Cancer is a worldwide disease that ranks as the second reason for death. Statistics show there are 1,688,780 new cases in the United States in 2017. It means 4600 new cancer patients appear every day. Figure 1 shows the trend in cancer incidence rates in the United States over the past few decades [1]. In the past decade, the incidence rates of lung cancers have declined annually, and the trend of decline is accelerating. Among them, lung cancer incidence rates in males declined about twice as fast as in females. Regardless of sexual distinction, lung cancer accounts for the top three cancer incidence rates. More than a quarter (26%) of cancer deaths are due to lung cancer. Although the survival rate of most cancers is steadily rising, the mortality rate of lung cancer has not declined. The five-year survival rate is only 18% [1]. Fortunately, the early diagnosis of lung cancer will greatly increase this ratio because it can potentially improve the prognosis [2].

Recently, the adoption of low-dose Computed Tomography (CT) has been widely used for lung cancer inspection [3]. Compared to other imaging techniques, CT achieves more advantages since it can visualize small or low-contrast nodules. Therefore, early detection of lung nodules on lung CT scans is of great significance for the successful diagnosis and treatment of lung cancer. 

Traditionally, clinicians observe, analyze, and interpret the lesion information according to the results of nodule morphology and clinical conditions [3]. However, there exist three main drawbacks. The number of cases is very large. The interpretation of each lesion by radiologists is a complicated process. In addition, its inefficiency may cause patients to miss the best time for treatment [4,5,6]. The diagnosis is subjective and the professional levels of clinicians are discrepant. Different clinicians may give diverse interpretations (this phenomenon has already been confirmed in the label of LIDC-IDRI, and this will affect the accuracy of diagnosis). Due to the clinician’s personal physical factors such as limitation of the human visual system, fatigue, and distraction, clinicians do not make the best use of the CT image data [7]. Therefore, an automatic mode is needed, which can help clinicians analyze CT images, reduce the workload, identify and exploit the nodule that even experienced chest radiologists may miss, and increase the accuracy of diagnosis [3]. 

**Figure 2:**
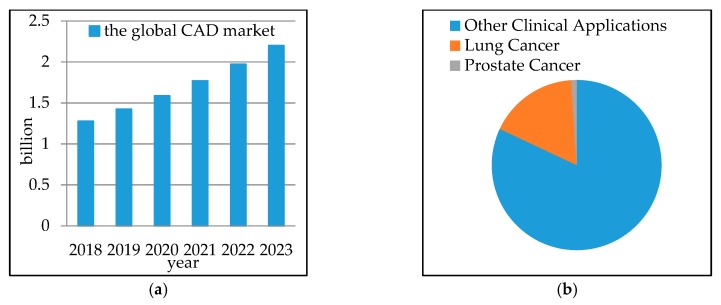
Demonstration of the CAD market trend and its market share of lung cancer. (**a**) prediction of global CAD market, (**b**) proportion of different CAD systems applications.

Computer-aided diagnosis (CAD) systems are proposed to assist clinicians in medical information interpretation and disease diagnosis. CAD can be divided into two types: detection system (CADe) and diagnostic system (CADx). The goal of CADe is to locate the interest regions of the image to find specific anomalies. The CADx system provides medical assistance for clinicians to distinguish the type, severity, stage, progression, and deterioration of disease. The CADx system can use only diagnostic-related data [8] (e.g., shape, density, and texture). For a lung cancer early diagnosis, a CAD usually consists of three stages: (i) data acquisition, (ii) nodule candidates segmentation, and (iii) nodule type classification. Among these stages, nodule type classification is the most important one since it can provide fundamental clues for clinicians to make a final decision. As an extension of CAD, radiomics extracts a large number of quantitative image features from a large number of patient data, and then analyzes various physiological processes [9]. By the year 2023, the global CAD market is estimated to reach $2.2 billion, with a compound annual growth rate (CAGR) of 11.5% in the forecast period, which is shown in Figure 2a. Figure 2b demonstrates that 23% of CAD systems are used for lung cancer [10]. This indicates that the diagnostic technology is increasingly accepted and rapidly applied.

For its key role in lung cancer diagnosis, this paper gives an appraisal of lung nodule automatic classification algorithms for CT images, which mainly focus on feature extraction and classifier construction. Related works are first retrieved from widely used academic datasets, including DBLP, Web of Science. Definition and formulation of nodule type classification are given. Two research objectives are listed, including four-type classification based on morphology and location, and two-type classification based on malignant rating. In addition, databases commonly used are explained in detail. Then, each work is reviewed and the corresponding method is described. Categorization and measurements are given for all literature based on the types of characteristics. Moreover, analysis is given according to method characteristic, performance, and the development trend.

The rest of the paper is organized as follows. Section 2 introduces the source and criteria for literature selection. Section 3 gives the problem statements, which describe the task of lung nodule classification. The required databases are explained in Section 4. Section 5 presents the main process of lung nodule classification and its metrics. The covered literature is analyzed in Section 6. The methods are listed and categorized. Section 7 disuses the limitation, challenge, and future development trend of this field. Lastly, Section 8 concludes this paper.

## 2. Work Selection Criteria

Works related to lung nodule CT image classification were selected, and articles from DBLP and Web of Science were covered up to June 2018.

This systematic review is made based on the following procedures: (i) relevant keywords are input into specific databases, and retrieval is performed, (ii) repeated works are removed, (iii) each work is grouped based on a set of defined indicators, such as lung nodule database used, feature extracted, and type of nodules.

The keywords, lung nodule classification, and pulmonary nodule classification were adapted to search engines of Web of Science, IEEEXplore, and DBLP. In the initial survey, 311 works were obtained. After identification and removing of the repeated one, 243 works were selected for the next step. After analysis and selection, 108 articles were identified as the target in this review.

## 3. Nodule Classification Statements

**Figure 3:**
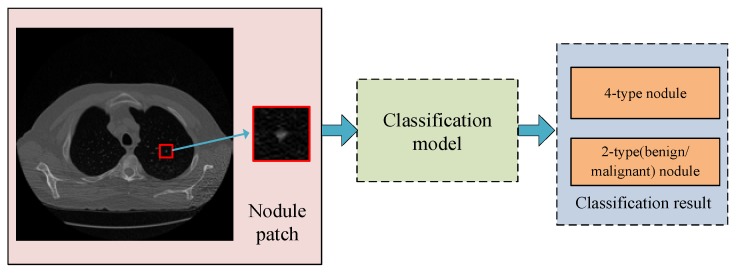
Problem statements of lung nodule classification in this research.

For nodule classification in lung CT images, there are mainly two research directions. (i) Four-type nodule classification (based on appearance, shape, and location), and (ii) two-type nodule classification (based on rating of malignant). Figure 3 gives the problem statements of lung nodule classification in our research. These two problems are explained in detail in this section.

### 3.1. Four-Type Nodule Classification

**Figure 4:**
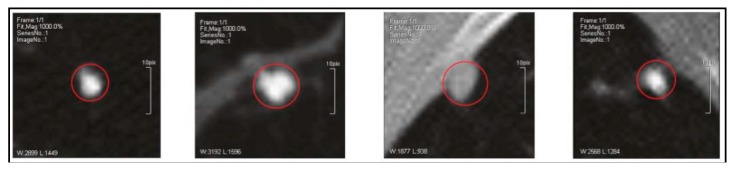
Demonstration of four types of lung nodule CT image, shown from left to right, W, V, J, and P, respectively [12]. Red circles denote the locations of the nodule.

Lung nodules are usually small and spherical masses inside lungs. Nodules can be distorted by neighboring anatomical structures, such as vessels and pleura. Therefore, the lung nodule is usually characterized by its appearance and surrounding regions. The definition from Reference [11] is the most popular approach and it divides nodules into four types: well-circumscribed (W), located in the center of the lung without any connection to other tissues, vascularized (V), similar as W except for connecting to vascular structures, juxta-pleural (J), fully connected to pleural surface, pleural-tail (P), close to pleural but only connected with a thin tail, as shown in Figure 4. 

Many works have been addressed to solve this problem. The input is a lung nodule image patch, and the output is the type. As formally shown in Equation (1), let *x* be a lung nodule image patch with size of l*w*c (*l, w*, and *c* denote the length, width, and channel). *y* is the nodule type. *f()* is the classification method.

(1) $$y=f\left(x\right)\;\left(x\in R^{l*w*c},y\in \left\{W,\;V,\;J,\;P\right\}\right)$$

### 3.2. 2-Type Nodule Classification 

**Figure 5:**
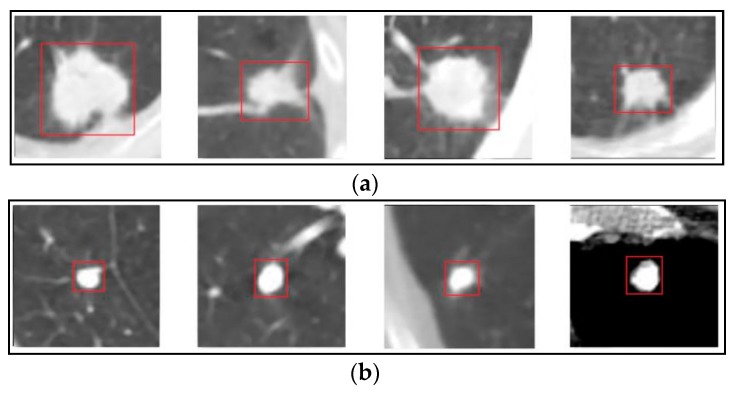
High malignancy suspicious cases are given in (**a**) and low malignancy suspicious cases are given in (**b**) [13]. Red bounding boxes denote the locations of the nodule.

There are many studies concerning the problem of two-type nodule classification (benign/malignant rating). It is also considered as the problem of lung nodule malignancy suspiciousness (the likelihood of nodule malignancy) classification. The formula is similar to Equation (1), where y denotes the nodule malignancy (benign and malignant are represented with 0 and 1). Nodule cases with high malignancy suspicious and low malignancy are shown in Figure 5.

## 4. Main Datasets 

For lung nodule image classification, the ground truth dataset is essential for training and testing designed models. Due to the policies and the privacy of patients, it is hard to obtain datasets as easily as in other fields. Therefore, some researchers use data from private databases or local hospitals. At present, some public datasets, including LIDC, LIDC-IDRI, ELCAP, JSRT, NELSON, ANODE09, and DLCST, are widely used for evaluating performance of different algorithms [14,15]. These datasets are introduced in this section.

### 4.1. LIDC and LIDC-IDRI

The Lung Image Database Consortium and Image Database Resource Initiative (LIDC-IDRI) consisted of diagnostic and lung cancer screening thoracic CT scans with marked-up annotated lesions. This dataset was initiated by the National Cancer Institute (NCI). Seven academic centers and eight medical imaging companies provided most of the data for early-stage lung cancer analysis [15]. 

The initial version of LIDC contained 399 scans. LIDC-IDRI expanded LIDC to 1018 scans of 1010 patients. For each CT scan, a two-stage image annotation process was performed with four experienced radiologists by using a marking approach similar to LIDC and an associated XML file for recording the results. In the first blinded-read stage, each radiologist independently read and marked each case. In the subsequent un-blinded-read phase, the results were compiled and sent back to the same radiologists. Each radiologist reviewed their own marks and the marks of the other three radiologists. The radiologists then read and annotated each case independently in order to make a final decision. Such a two-phase labeling can identify all lung nodules as completely as possible, avoiding forced consensus [15]. The region of the lesion belonged to three categories: (i) nodules >= 3 mm, (ii) nodules < 3 mm, and (iii) non-nodules >= 3 mm.

**Figure 6:**
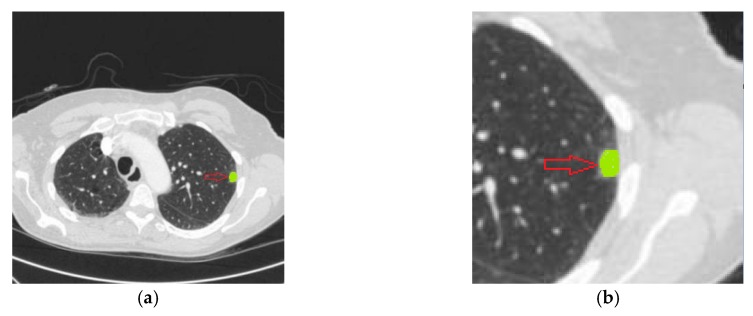
Lung CT image [13]: (**a**) Original image with nodule (green color), (**b**) the part of CT image with nodule > 3 mm ROI (green color). Note that the xml file includes the outline of the node (only its boundary points). In this case, the entire nodule is displayed for better understanding and visibility.

Lastly, the results from each of the four radiologists were combined into an xml file for a specific CT series. It contained descriptions of all nodules > 3 mm (contours and subjective assessments of nodule characteristics), the approximate 3-dimensional center-of-mass of nodules < 3 mm, and all non-nodules (no contour or subjective assessments of characteristics). Totally, the LIDC-IDRI contained 7371 lesions marked “nodule” with at least one radiologist, and 2669 lesions of these were marked by at least one radiologist as nodule >= 3 mm. An example image with displayed markings is provided in Figure 6. Table 1 presents the frequently used nodule description.

Each row indicates the relevant information of a nodule. The first column is a part of the patient ID. The second column indicates the unique nodule identifier. The third and fourth give the x and y position of the nodule. The Z location describes the location of the slice where the nodule is contoured. The sixth column means the collection of (x, y) points that make up the boundary of the nodule (only a part of coordinates are given in this table). The last column means malignancy of this nodule rating by radiologists. The upper two rows describe the information (location, contours, and assessments) of nodule >= 3 mm and the others describe the information (location) of nodule < 3 mm.

### 4.2. ELCAP Public Lung Image Database

The Early Lung Cancer Action Program (ELACP) database was first released in December 2003. This database was made by collaboration between ELCAP and VIA research groups, which was usually used to evaluate performances of different CAD systems [12,16].

The database consisted of 50 low-dose documented whole-lung CT scans and 379 unduplicated lung nodule CT images. The CT scans were obtained in a single breath hold with 1.25 mm slice thickness. The locations of nodules detected by the radiologist were also provided.

**Figure 7:**
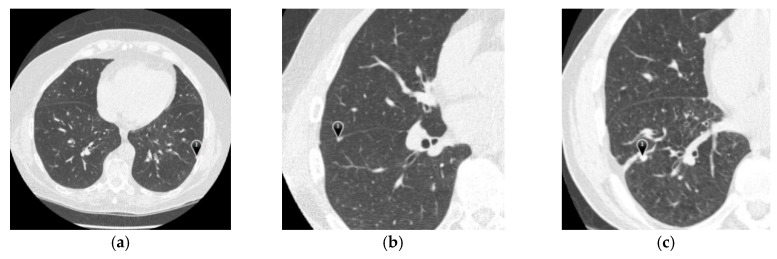
Demonstration of lung CT images from ELCAP [12]: (**a**) the complete CT images, (**b**,**c**) are the part of CT scans. The symbol “1” is the location of nodule.

The percentages of nodule types in ELCAP were W-15%, V-16%, J-30%, and P-39%, respectively [17]. ELCAP was different from LIDC-IDRI in two aspects: its nodule sizes were relatively small and non-nodules were not included [18]. Each case had an extra *.csv file containing the center position of all identified nodule, which is shown in Table 2. Each row indicates a lung nodule. The first column is the identifier of scan. The third and fourth columns give the center position of lung nodule. The last column describes the location of this slice on which the nodule exists. Figure 7 demonstrates lung CT images from ELCAP.

### 4.3. Others

The JSTR database was constructed by the Japanese Society of Radiological Technology in cooperation with the Japanese Radiological Society. The database contained 154 nodules and 93 non-nodules with labels [19]. The additional information included patient age, gender, subtlety degree, nodule size, and its coordinates. 

Nederland-Leuvens Longkanker Screenings Onderzoek (NELSON) collected data of 15,523 participants in four institutions since 2003. Annual CT screening were interpreted at local institution and then again at a central site. They provided resources, i.e., semi-automated lung nodule volume measurement, discrimination between benign and malignant nodules, automated lung nodule detection, and segmentation [20].

Automatic Nodule Detection 2009 (ANODE09) database consisted of five example and 50 test scans [21]. ANODE09 provided a platform for nodule detection algorithm comparison. Any team can upload their results for benchmark evaluation [22]. In some literatures, researchers employed databases collected from private hospitals or other organizations. These databases are less used and there is no detailed description. Table 3 gives a brief explanation for these databases.

Among the above databases, LIDC-IDRI and ELCAP were the most popular ones. The LIDC-IDRI was mostly used for the two-type nodule classification of benign and malignant, while the ELCAP was mostly employed for the four-type nodule classification. 

## 5. Main Process Introduction 

### 5.1. Feature Extractions and Selection

Lung nodules are low-contrast tissues that cannot be easily distinguished. Their main characteristics are represented by features vectors, extracted from lung nodule image, based on their shapes, texture, densities, intensity values, fractals, and deep features. These features may be either 2D or 3D and sometimes both are used.

Some selected papers are based on user-defined features, including shape [29,30,31,32,33,34,35,36,37,38,39,40,41,42,43,44,45,46,47,48,49,50,51,52,53,54,55,56,57,58,59,60], texture [29,33,34,35,36,37,38,39,41,44,45,46,47,48,50,54,59,61,62,63,64,65,66,67,68,69,70,71,72,73,74,75,76,77,78,79,80,81,82], size [33,36,39,48,77,83,84,85], density [55,56,57,58,79,86,87,88,89,90], morphological [42,54,60,62,77,82,88,91,92], statistical [18,39,46,50,57,93,94,95,96,97], geometric [40,53,69,73,81,88,93,94,96], intensity [18,29,30,36,37,39,50,53,56,59,67,70,71,73,79,83,97,98], grey level [38,39,42,53,60,77,88,94,99,100,101], boundary [24,41,86,99,102,103], curvature [56,57,58,90,104], wavelet [36,37,62], context [43,50,70,79,102,105], patient characteristics [48,54,91], attenuation [33,48,59,101], growth changes [106], and semantic features [74].

Some selected papers are based on generic features, including scale-invariant feature transform (SIFT) [17,70,71,72,96,107], local binary pattern (LBP) [41,70,73,74,76,107], histogram of oriented gradient (HOG) [43,50,70,94,107], Gabor [39,41,72,73,74,107], and speeded-up robust features (SURF) [76,99].

Selected papers based on deep feature are given in References [18,42,43,44,45,60,77,83,92,95,96,103,105,108,109,110,111,112,113,114,115,116,117,118,119,120,121,122,123,124,125,126,127,128,129]. Selected papers that based on 3D features are given in References [18,46,47,48,49,50,51,52,53,54,55,56,57,58,59,60,78,79,80,81,82,83,88,89,90,96,97,98,105,110,123,124,125,126,127,128,129,130]. 

### 5.2. Classifier

The classifier is the model that can distinguish feature vectors of different nodule types. The main methods for classifier construction are machine learning-based, which uses the training algorithm and labeled data. Many kinds of classifiers are used in selected papers, such as those based on support vector machines (SVM) [29,34,35,36,37,39,41,46,47,49,62,63,64,65,70,71,75,80,81,92,94,96,99,100,101,102,107,110,115,130], bayesian classifier [35,40,46,62,98,117], rule based [106], k-means [57,58,86], linear discriminant analysis (LDA) [33,46,54,57,58,68,72,76,82,88], artificial neural networks (ANNs) [44,45,50,55,62,66,69,77,78,79,104,112,121,122], convolutional Neural Networks (CNNs) [18,29,42,60,83,103,105,108,109,111,112,113,114,118,120,121,123,124,125,126,127,128,129], k-nearest neighbors (KNN) [29,41,51,52,53,62,78,85,94,107], fuzzy system [67], random forest (RF) [29,30,38,43,62,73,78,86,94,97,110,125], spectral clustering algorithm [61], decision tree [39,107,119], deep belief networks (DBNs) [120], stack auto-encoder (SAEs) [112,116,120], probabilistic latent semantic analysis (pLSA) [70], linear classifier [59,93], nonlinear classifier [93], logistic regression [48,84,85,94,131], minimum distance classifier [107], semi-supervised [31,132], weighted Clique Percolation Method [17], ensemble classifier [46,77,83,91,94,107,133], random subspace method (RSS) [91], and content-based image retrieval (CBIR) [56,74,87,90].

### 5.3. Measurement

For a lung nodule classification algorithm or system, the performance is evaluated by the following measures.

(2) $$\mathrm{Sensitivity}:\;\mathrm{Sen}\;=\;\frac{\mathrm{TP}}{\mathrm{TP}+\mathrm{FN}}$$

(3) $$\mathrm{Specificity}:\;\mathrm{Spe}\;=\;\frac{\mathrm{TN}}{\mathrm{TN}+\mathrm{FP}}$$

(4) $$\mathrm{Accuracy}:\;\mathrm{Acc}\;=\;\frac{\mathrm{TN}\;+\;\mathrm{TP}}{\mathrm{TP}\;+\;\mathrm{TN}\;+\;\mathrm{FN}\;+\;\mathrm{FP}}$$

Equations (2)–(4) present the Sensitivity, Specificity, and Accuracy, respectively. Where TP (true positive) stands for the number of nodules that are correctly identified. FN (false negative) is the number of nodules classified as negative but actually are positive. TN (true negative) represents the number of nodules classified as negative and actually are negative. FP (false positive) is the number of nodules classified as positive but actually are negative.

Sensitivity means how well the algorithm recognizes the type of nodule correctly, and a high sensitivity value represents a low rate of a missed diagnosis. Specificity measures the ability of the algorithm to remove the false type of nodule, and a high specificity value means a low rate of misdiagnose. Accuracy measures the proportion of data that correctly classified. Sensitivity-specificity ROC curve is often used to characterize the performance of the classifier at various operating points. The area under the curve (AUC) is another indicator used to evaluate the performance of a classifier.

## 6. Analysis of Selected Work

This section describes the methodologies employed in the selected works. They are grouped, according to the types of features: user-defined features, generic features, deep features, 3D nodule features, and other features. Because feature representation engineering plays the leading role, we pay more attention on the lung nodule feature design instead of the classifier. The following subsections give the detailed description.

### 6.1. User-Defined Features 

User-defined features, such as texture, shape, intensity, and fractal, are mostly designed by experienced scientists and engineers through quantitative analysis. They are covered in References [18,29,30,31,32,33,34,35,36,37,38,39,40,42,43,44,45,46,47,48,49,50,51,52,53,54,55,56,57,58,59,60,61,62,63,64,65,66,67,68,69,75,78,79,80,81,82,84,85,86,87,88,89,90,91,92,93,94,95,96,97,98,99,100,101,102,103,104,105,106,125,130,131,134].

An unsupervised spectral clustering algorithm was used in Reference [61]. The Laplacian matrix for capturing the discriminative information was created by using local kernel regression models (LKRM) and a linear regression. The nodule was represented by Haralick texture features. Furthermore, 375 malignant and 371 benign nodules were collected from LIDC-IDRI. In addition, 10.9% of malignant nodules and 17.5% of benign nodules were mistakenly classified.

Two novel experiments were implemented in Reference [93]. The first was to determine the learning capability of statistical learning algorithms. The second was to determine whether increasing features can improve the linear/nonlinear classifier’s ability, and which diagnostic features were more useful. After excluding inconsistently cases, 2817 annotations from LIDC-IDRI were used. The nonlinear classifier obtained an accuracy of 85.74 (±1.14)% and an AUC of 0.932 (±0.012). When including diameter and volume features, the AUC increased to 0.949 (±0.007) with an accuracy of 88.08 (±1.11)%. 

In Reference [86], a large number of blocks from lung tumor images were randomly collected, and the distance matrices were generated by calculating the relationships among blocks. K-means clustering was then applied and density distribution features were calculated. The Random Forest was used for identifying lung adenocarcinoma risk levels. The technique achieved the AUC of 0.9144 ± 0.0411 (*p* = 0.0002) and the best accuracy of 89.20% on the ZSDB database. When evaluated on LIDC-IDRI, the technique obtained the AUC of 0.8234 ± 0.0703 (*p* = 0.0009) and the best accuracy of 82.92%. 

CBIR was used for lung nodule classification in Reference [87]. For each nodule, they used two-step CBIR scheme to retrieve the similar nodules and a probability value was calculated for classification. The LIDC-IDRI for evaluation was divided into two datasets. The model obtained an AUC of 0.751 and the accuracy of 71.3% when using the second dataset to retrieve the first dataset.

Performances of various classifiers were evaluated in Reference [29,62,94]. First, features based on shape, geometric, histogram, and texture were extracted. Then multiple classification algorithms were implemented for testing and the one with best performance was adopted.

Ma and Wang [30] proposed an automatic nodule classification technique based on radiomics approach. Furthermore, 583 features were used for representation, including shape, intensity, and multi-frequencies heterogeneity. RF was used as classifier for classifying benign and malignant nodules. This technique used 79 CT scans from The Cancer Imaging Archive (TCIA) containing 127 nodules annotated by at least one radiologist for evaluation and achieved 82.7% accuracy.

Song and Liu [31] proposed a slightly supervised method based on the Partial Label Error-Correcting Output Codes (PL-ECOC) algorithm for nodule classification. Eleven shape-based features were extracted. Additionally, 188 CT scans provided by the LIDC and Qianfoshan Hospital were used. The method achieved an accuracy of 83.4%. 

Huang and Tu [131] presented a logistic regression classifier for classifying malignancy of lung nodules. A total of 238 features were extracted from open research platform of radionics (IBEX). A forward search algorithm was applied for selecting features and nine features were identified. This system was evaluated with 100 CT image series and it achieved the accuracy of 79% and AUC of 0.81 ± 0.01.

In Reference [91], the morphological feature, demographic feature (age, gender), contrast, localization, and nodule diameter were extracted and 10 features were selected. With CT images of 35 different patients, this method got 89.5% accuracy, and sensitivities of 94.7%, 90.0%, and 77.8% for benign, malignant, and uncertain classes.

In Reference [63], the histogram equalization was used for preprocessing. Shannon and Simpson’s indices were applied as texture descriptors for lung nodules. LDA was used for feature selection and SVM was trained for nodule classification. In addition, 73 nodules (26 malignant and 47 benign) from LIDC were used for evaluation. It had the sensitivity, specificity, and accuracy of 85.64%, 97.89%, and 92.78%, respectively.

In Reference [32], a private dataset contained 102 high-resolution CT (HRCT) images of lung nodules, which were collected from 102 patients. Circularity and the second moment were used as a feature representation.

McNitt-Gray and Har [33] employed semi-automated contour to segment nodule regions. Then attenuation, size, texture, and shape features were extracted based on the contours. Two different texture measures, correlation, and entropy were selected as the input of LDA to distinguish benign and malignant nodules. Hence, 90.3% accuracy was gained on 31 testing cases. 

Song and Stephanie [64] used various texture features based on dual time F-fluorodeoxyglucose (FDG) PET/CT images (DTPI). Sequential forward floating selection (SFFS) was performed for critical feature selection to train the SVM classifier. Features with wavelet transform, convergence index filter, LOG, and Adaboost were extracted in Reference [65]. SVM was used as a classifier and this method was tested on JSRT.

Dhara and Mukhopadhyay [34] used a semi-automated technique for nodule segmentation. Then shape and texture features were extracted. An AUC of 0.9465 was achieved by using the SVM classifier when used as a set of 542 nodules from LIDC-IDRI for evaluation.

A novel feature Local Difference Pattern (LDP) was extracted, according to the gray level difference between lung nodules and its neighbor regions [99]. The single-center classifier was first constructed by using SVM and LDP. The multi-center classifier was constructed based on unsupervised method. These two classifiers were combined to build the final decision. ELCAP was used for evaluation and a more than 90% classification rate was obtained.

In Reference [35], segmentation was performed by thresholding and morphological operations. Then, the regions of interest (ROIs) were generated by using priori information and Hounsfield Units (HU). Shape and textural features were calculated and SVM was trained for classification. SPIE-AAPM Lung CT Challenge dataset with 70 thoracic CT scans was used for evaluation and 78.08% accuracy, 84.93% sensitivity, and 80.92% specificity were obtained.

A quantitative radiomic method was proposed by Reference [36]. Based on radiomic analysis, 150 features were extracted to quantify nodule image texture, intensity, and shape. RF was used for feature selection. In addition, 15 defined features were selected and SVM was trained for classification. The genetic algorithm (GA) was used to optimize parameters and 76.1% accuracy was gained. Similar methods were proposed in References [37,38]. Segmentation as the first step was incorporated in Reference [37].

Aggarwal and Vig [39] combined shape, texture, size, resolution informatics, statistical and intensity features descriptor, and principal component analysis (PCA) was performed for feature selection. They achieved accuracy of 82.32% for patient-wise data. 

Lin and Huang [100,101] used a set of fractal features based on fractional Brownian motion model. In addition, 107 CT images were collected from 107 patients for evaluation. The method obtained an AUC of 0.8437, accuracy of 83.11%. In Reference [101], the accuracy, sensitivity, specificity, and the AUC were 88.82%, 93.92%, 82.90%, and 0.9019 when combining the CT attenuation value with a fractal-based method.

The lung nodule and the surrounding context information were used for feature representation in Reference [102]. Patch was labeled as foreground and background with Superpixels. Intra-features and inter-features of the nodule were constructed. The SVM classifier was trained for classification tasks. Fifty lung CT scans with 379 nodules from ELCAP were used. This method obtained an accuracy of 82.5%.

The Gray level Co-occurrence Matrix (GLCM) was employed in References [66,67,68]. An Artificial Neuro-Fuzzy Inference System (ANFIS) was proposed in Reference [67]. Morphological operation was applied for segmentation. GLCM was used to extract texture features and then were standardized by PCA. ANFIS was used to train the model. The algorithm was validated on 617 nodules of 151 CT scans from LIDC-IDRI and SPIE-AAPM database. The system presented the sensitivity of 98% for finding true lung nodules, the sensitivity and accuracy of 94.4% and 85% for removing false detection, and the sensitivity and accuracy of 87% and 78% for cancerous nodule classification. Similar methods were proposed in References [40,69], which included morphological aspects for preprocessing and features of shape that were extracted.

In Reference [66], neural networks model of Self-Organizing Maps (SOM) was provided for the segmentation of nodules. GLCM and PCA were used. A total of 128 CT images from 47 patients contained 128 nodules (52 malignant and 76 benign) with 50% of the data for training and the other 50% for testing. The system using ANNs achieved the sensitivity, specificity, and accuracy of 92.30%, 89.47%, and 90.63%, respectively.

McNitt-Gray and Wyckoff [68] used a linear discriminant classifier (LDC) to evaluate the performance of the different combinations of texture features for classification. It obtained the AUC of 0.992 and accuracy of 93.8% when using the same data for training and testing, while an accuracy of 90.6% was reached when using jackknifing.

In addition, a two-level ANN was trained as a classifier and curvature features were used as input [104]. Kim and Park [84] developed a semi-automatic diameter measurement method to improve the diagnostic accuracy. Jia and Bai [106] extracted the growth changes of nodules by combining the global rigid registration with local elastic registration method and classified benign and malignant nodules with a rule-based classifier. Jirapatnakul and Reeves [85] made an experiment to determine the effect of distribution of different sizes on the performance of nodule classification system. Logistic regression and a k-NN classifier were applied.

### 6.2. Generic Features

Well-engineered feature extraction and representation methods widely used in the computer vision domain were adopted in lung nodule image classification. These generic features such as SIFT, SURF, HOG, LBP, and Gabor filters etc. were adopted in References [17,41,43,50,70,71,72,73,74,75,76,94,96,107]. 

SIFT was used in References [17,70,71,72]. Zhang and Song [70] developed a method for four types lung nodule classification based on contextual analysis. An adaptive patch-based division was generated and concentric multilevel (level-nodule and level-context) partitions were constructed. Then a feature set including SIFT, HOG, and MR8+LBP was extracted. A contextual latent semantic analysis-based classifier including SVM and Probabilistic Latent Semantic Analysis (pLSA) analysis was designed to calculate probabilistic estimations. A total of 379 nodules from the ELCAP were used for evaluation. 

Nodule type overlapping was discussed in Reference [17]. Probability estimates on the nodule type were calculated by the SVM with SIFT. A four-length vector was generated by projecting the SIFT descriptor for the representation of the nodule. A nodule similarity network was constructed with the probability vectors. Weighed Clique Percolation Method (CPMw) was used for the overlapping analysis. The method was evaluated on the ELCAP and had the average precision of 91.6%.

An optimized graph model based on conditional random field (CRF) was proposed in Reference [71]. The standard unary, pairwise terms, global, and region-based energy terms were designed to label the voxels as foreground or background. The location information was inferred by using a learning-based context characterization based on voxel labeling outputs. The SIFT features were extracted and the SVM classifier was trained. The algorithm showed effective performance on ELCAP. In Reference [72], the SIFT descriptor was applied for feature extraction. PCA and LDA were used for reducing dimensions. Gabor wavelet responses were obtained from an adopted Daugman Iris Recognition algorithm. The nearest-neighbor classifier was trained for classification. Furthermore, 294 nodules from ELCAP were used for evaluation. When the training percentage was 75%, the best accuracy of 78.23% was obtained.

In Reference [73], an anisotropic non-linear diffusion filter was proposed to remove noises and preserve boundaries. A random walk method was used for segmentation. Intensity, texture, and geometric features based on the GLCM, Gabor filter, and rotation invariant uniform LBP were extracted, and mutual information was used to reduce the dimensions. Lastly, an improved random forest classifier was trained. The proposed method achieved a sensitivity of 92% and AUC of 0.95 when evaluated on LIDC-IDRI. Mean sensitivity and specificity were 85% and 82% when evaluated on the General Hospital of Guangzhou Military Command dataset.

Similar to Reference [87], CBIR was adopted in Reference [74]. LBP, Gabor feature, and Haralick features were used as representation. This algorithm proposed a new two-step CBIR scheme (TSCBIR) with two similarity metrics, semantic relevance, and visual similarity, which measured the similarity of different nodules. Distance metric learning was used for similarity metrics. Furthermore, 366 nodule ROIs (191 malignant, 175 benign nodules) were obtained from LIDC-IDRI. In this work, nodules with the rating > 3.5 were labeled as malignant, and nodules with the rating < 2.5 were labeled as benign. The algorithm obtained an AUC of 0.986 and classification accuracy of 91.8%.

In Reference [41], feature fusion based on Gabor filter, multi-resolution LBP, and shape texture were used. SVM and kNN were applied for the classification of benign, malignant, or non-nodule. A total of 1191 nodules and non-nodules from LIDC with a diameter between 3 and 10 mm were used for analysis. Nodules of malignancy level 3 were not considered. The results from the two-tier cascaded SVM with Gabor feature showed an average AUC of 0.99 and average f1-score of 0.975.

In Reference [75], the active contour method was first used to segment the lung regions and nodules, which were extracted by calculating eccentricity. GLCM was used to calculate the Haralick texture features. SVM was employed to classify the nodules into benign or malignant. The approach obtained the accuracy of 92% on LIDC-IDRI.

Mao and Deng [107] developed a nodule classification technique by using an ensemble classifier set with different feature extractions and representations. The lung nodule feature was represented with SURF and LBP descriptor in Reference [76]. PCA and LDA were used for subspace projections. The method was evaluated in ELCAP and the experimental results showed that the LDA-LBP classification obtained the best overall classification results. When the training percentage was 75%, the best accuracy obtained was 81.5%.

### 6.3. Deep Features

With the increase of the training datasets and the improvement of computational power, deep learning has achieved great success in the field of machine learning, especially in the field of image classification. Deep learning forms cascaded multilevel nonlinear processing units to construct deep structures for feature extraction and representation. The parameters in the deep model can be automatically adjusted. Deep learning models, such as CNNs, Auto-encoder, DBNs et al. were used in lung nodule image classification [18,42,43,44,45,60,77,83,92,95,96,103,105,108,109,110,111,112,113,114,115,116,117,118,119,120,121,122,123,124,125,126,127,128,129].

Zhao and Liu [108] proposed Agile CNNs for lung nodule classification. The model was constructed on the basis of LeNet and AlexNet. It contained 7 × 7 kernels with only two convolutional layers. The model was trained and tested with 743 CT images (368 benign and 375 malignant nodules) from LIDC-IDRI. It achieved the accuracy of 82.2% and AUC of 0.877.

Multi-group patches were used in Reference [109]. Segmentation and enhancement were performed. A four-channel CNN with original and binary images as input was designed. This method was evaluated on LIDC-IDRI.

A Convolutional Deep Belief Networks (CDBNs) model was proposed in Reference [111]. Three convolutional restricted Boltzmann machine (CRBM) layers were trained by unsupervised methods. A softmax classifier was trained for two-type nodule classifications. The algorithm was evaluated on 1200 benign and 1000 malignant nodules from LIDC-IDRI, with the ROI size of 38 pixel × 38 pixel. The model got the best classification accuracy, precision, recall, and F1 value of 92.83%, 90%, 97.30%, and 93.51%, respectively. 

Three types of models known as CNNs, DNNs, and SAEs were analyzed in Reference [112]. The CNNs contained two convolutional layers, two max pooling layers, two fully connected layers, and a softmax layer. The DNNs contained four fully connected layers and a softmax layer. The SAEs contained three fully connected layers and a softmax layer. In addition, 9106 nodule images were collected from LIDC-IDRI. According to the degree of malignancy, nodules with scores of 1 and 2 were identified as benign, and scores of 4 and 5 were identified as malignant. The size of the nodule images was set to 28-pixel × 28-pixel. The CNNs model achieved the best performance with an accuracy, sensitivity, and specificity of 84.15%, 83.96%, and 84.32%, respectively.

Da Silva and da Silva Neto [113] suggested an approach for benign and malignant nodule classification by using CNNs jointly with a genetic algorithm. Nodule regions were first segmented with Otsu. Multiple CNNs models were designed with nodules and its sub-regions by shared, fully connected layers. The genetic algorithm was used to optimize the parameters. Nodules with rating 1 and 2 were labeled as benign, rating 4 and 5 as malignant, and rating 3 as indeterminate. The method was validated on 3243 nodules (1413 malignant nodules and 1830 benign nodules). The sensitivity, specificity, accuracy, and AUC of 94.66%, 95.14%, 94.78%, and 0.949% were achieved.

In Reference [114], a multi-view CNNs method was proposed for nodule classification. Seven different views were cropped and a softmax classifier was trained. After data augmentation, 27,504 benign and 28,240 malignant nodules from LIDC-IDRI were used. For binary classification (benign and malignant), an error rate of 5.41% was achieved. For ternary classification (benign, primary malignant, and metastatic malignant), an error rate of 13.91% was achieved. 

The method in Reference [103] adopted curriculum learning, transfer learning, and deep residual learning for improving classification performance. A fixed linear transformation was used to normalize the voxel values. Three orientations (axial, sagittal, and coronal) of extracted planes were considered. Three ResNet-18 with full convolution instead of fully connected layer were used. The final classification results were obtained by a weighted sum of network outputs. The model was evaluated on LIDC-IDRI and presented an accuracy of 89.90%.

Xu and Zhang [115] proposed a hybrid model named DGnet-SVM, which integrated GoogLeNet, Denoise Network, and SVM to classify benign and malignant nodules. The Denoise Network was used for enhancing the image quality. A fusion input strategy with noisy images with de-noised images was designed for improving the robustness. GoogleNet was employed for extracting deeper features, and SVM was trained for classification. A total 742 benign samples and 553 malignant samples from LIDC-IDRI were selected. The model was trained with 2590 augmented samples. The method had the accuracy of 89% and AUC of 0.95.

Jia and Zhang [116] applied the stacked generalization principle and SAEs for classifying lung nodules. Thammasorn and Wu [117] explored a multi-band network that used modified CNNs with a triplet network for feature extraction to cope with images of variable size and orientation. They used a retrospective analysis of 194 cases. The accuracy of different learning algorithms using texture, shape, and CNNs features was compared. The multi-band network was similar in accuracy (80.2%) to the texture and semantic shape features. 

Models of DBNs and CNNs were used in Reference [118]. A total of 2545 nodules from the LIDC-IDRI were selected with a diameter larger than 3 mm. The nodule ROIs were resized to 32 pixel × 32 pixel as input. The classification was performed through a combination of unsupervised pre-training and subsequent supervised fine-tuning. Lastly, DBNs achieved 73.4% sensitivity and 82.2% specificity, and CNNs obtained the sensitivity of 73.3% and the specificity of 78.7%. 

In Reference [119], nodule features were extracted from an auto-encoder (five layers for de-noising auto-encoder trained by L-BFGS) along with a binary decision tree as a classifier. A total of 4323 nodules from LIDC-IDRI were used. The method obtained the accuracy of 75.01% and the sensitivity of 83.35%.

Mao and Tang [95] used local and global features for nodule representation. Deep anto-encoder was employed for transforming local patches obtained by the super-pixel into local features. The bag of visual words (BOVW) was applied to generate global features. The softmax was used for the four-type classification task. The method was evaluated on ELCAP and reached the best accuracy of 93.9%.

Xie and Xia [42] adopted transfer learning for lung nodule classification. The ensemble model was constructed with three ResNet-50 for characterizing the appearance, voxel, and shape. The final results were obtained by weighting these models. In addition, 1357 nodules (873 benign and 484 malignant) were collected from LIDC-IDRI. The algorithm showed a classification accuracy of 93.40%.

Chen and Qin [43] studied the mapping between computation features and semantic features. The computation features included features extracted from SDAE and CNNs, HOG, and hand-crafted Haar-like features. Multi-task learning were adopted. 

Xie and Zhang [44] used the CNNs model with three convolution layers, three max-pooling layers, and three full connected layers. GLCM and the Fourier descriptor was used for extracting the texture and shape features. A back propagation neural network (BPNN) with three layers was trained by the 132-dimensional combined features. The algorithm was evaluated on 1181 benign and 387 malignant nodules from LIDC-IDRI, obtaining the accuracy, sensitivity, and specificity of 86.79%, 60.26%, and 95.42%, respectively.

A stacked de-noising auto-encoder based approach was developed for lung nodule classification [92]. It used three hidden layers to extract 100 features, and these features were combined with 96 hand-crafted morphological features. A simple *t*-test was performed for a feature selection. Lastly, the SVM classifier was trained. To train and test the approach, 3598 nodules (178 malignant and 3420 benign nodules) were collected. The approach achieved an accuracy, sensitivity and AUC of 95.5%, 94.4%, and 0.987, respectively.

CNNs, DBNs, and SDAE models were tested in Reference [120]. The CNNs contained three convolutional layers and three pooling layers. The DBMs were constructed by four layers of RBM in a greed fashion. The SDAE had three layers. After data augmentation, 114,728 samples (54,880 benign and 59,848 malignant) resized to 52 pixel × 52 pixel from LIDC-IDRI were used. The accuracies of CNNs, DBNs, and SDAE were 79.76%, 81.19%, and 79.29%, respectively.

A fusion method was proposed in Reference [45]. Features of texture, shape, and deep model were calculated from a GLCM, Fourier shape descriptor, and 8-layer DCNNs. An Adaboosted BPNN was trained, and the result was made by three classifiers at a decision level. Additionally, 1972 nodules (1324 benign, 648 malignant, nodules of malignancy rate 3 were discarded), 2021 benign, and 648 malignant (regarding nodules with a malignancy rate 3 as benign), 1324 benign, and 1345 malignant (regarding nodules with a malignancy rate 3 as malignant) were considered. This algorithm achieved an AUC of 0.9665, 0.9445, and 0.8124, respectively.

In Reference [121], the methods of massive-training artificial neural networks (MTANNs) and CNNs were evaluated. Two conditions were considered in this study. In the first condition, the performance of CNNs and MTANNs was compared with limited training data. The results showed that MTANNs performed better than CNNs. In the second condition, they used a large training data set to train CNNs. There was a lower performance gap between two models. For nodule classification, 76 lung cancers and 413 benign nodules were used. MTANNs had an AUC of 0.8806, which was significantly greater than the best performing CNNs with an AUC of 0.7755.

In Reference [77], Canny’s edge detection technique was used for nodule segmentation. Fifty features including gray-level, texture, shape, and size were selected. The neural networks ensemble scheme was constructed by a multilayer neural network, a radial basis probabilistic neural network, and a learning vector quantization neural network. The outputs of individual neural networks were generated in the form of Bayesian probability and the Bayesian criterion was employed for the weighted sum of these values. Forty-seven nodules (9 benign, 14 uncertain for cancer, and 24 malignant) were used. Therefore, 78.7% accuracy was obtained, which was higher than the individual classifier.

In Reference [122], six MTANNs were trained simultaneously. The final probability result was computed by integrating each MTANN. The system used 76 malignant nodules in 73 patients and 413 benign nodules in 342 patients and achieved the AUC of 0.882.

### 6.4. 3D Image Based Features

3D features can utilize contextual and spatial information, and a better result can be achieved with these comprehensive features. In recent years, 3D-based features were commonly used to represent the lung nodule image. They were adopted in References [18,46,47,48,49,50,51,52,53,54,55,56,57,58,59,60,78,79,80,81,82,83,88,89,90,96,97,98,105,110,123,124,125,126,127,128,129,130].

Multi-view multi-scale CNNs based methods were proposed in References [18,96]. In Reference [18], a regularized sphere divided by icosahedra was constructed at the nodule center, and the volume was sampled by a concentric circle plane at a given radius. Data was resampling and high frequency content was computed to sort views. Lastly, CNNs were pre-trained with selected views at all scales and then retrained in a multi-view fashion. In addition, 1738 nodules and 1000 non-nodules with a 64 mm × 64 mm × 64 mm 3D volume were obtained from LIDC-IDRI for training and testing. Furthermore, 421 nodules were collected from ELCAP for testing. The method had the overall classification rate of 92.3% on LIDC-IDRI and 90.3% on ELCAP.

On the basis of Reference [18], Yuan and Liu [96] used multi-view multi-scale CNNs to extract statistical features after resampled views, and SIFT was extracted as geometric features. A multiclass SVM with multiple kernel learning was trained as classifier for types W, V, J, P, ground-glass optics (GGO), or non-nodule (N). Almost 3860 training and 1000 testing cases on LIDC-IDRI were applied. This method achieved an overall classification rate of 93.1% on LIDC-IDRI and 93.9% on ELCAP. A similar method was proposed in Reference [123] in which a multi-crop pooling layer was used as a substitution for the max-pooling layer to improve the classification performance. The method was evaluated on 880 benign and 495 malignant nodules from LIDC-IDRI and achieved the classification accuracy 87.14% and AUC 0.93.

In Reference [46], the region of the lung nodule was first segmented on CT images. After nodule segmentation, a set of 66 3D image features was computed to represent the density, shape, and texture of nodules. The multi-feature fusion based classifiers based on SVM, naïve Bayes classifier, and linear discriminant analysis were trained. Synthetic Minority Oversampling Technique (SMOTE) algorithm was applied to balance the training dataset. In addition, 243 nodules (76 benign, 81 stage I, and 86 stage III malignant) from Shanghai Pulmonary Hospital and NSCLC-Radiomics database were used. Three classifiers obtained the AUC with 0.94, 0.90, and 0.99, respectively. This study also confirmed that the AUC values of three classifiers were consistent in the same training data set. 

In Reference [130], thresholding and morphological operations were used to extract nodule candidates in 2D images, and then these candidates were stacked to structure 3D image. The region of the 3D image was manually cropped. The geometric features from the cropped 3D nodule were extracted and fed into SVM for classification. Thirty-one cases from TCIA were used. The algorithm achieved an accuracy of 90.9%. 

In Reference [47], a semi-automated technique was first used to segment lung nodules. A combination of 2D shape-based, 2D texture-based, 3D shape-based, 3D margin-based, and 3D texture-based features was calculated to represent lung nodules. SVM was employed for nodule classification. The approach was evaluated on 891 nodules from LIDC-IDRI for three configurations (score of malignancy 1 and 2 as benign, 4 and 5 as malignant in all configurations, 3 was considered benign, malignant, or discarded, respectively), which achieved AUC of 0.9505, 0.8822, and 0.8488, respectively.

2D and 3D radiological quantitative features were compared in Reference [48]. More than 50 quantitative features including size, shape, attenuation, texture, and margin were extracted in 2D from a single slice and in 3D from the entire nodule volume. Multivariable logistic regression was applied for classifying lung nodules into lung cancer that is metastatic or benign. 96 solid nodules from 94 patients were used. The researchers concluded that there was no significant difference between 2D and 3D analysis for distinguish primary lung cancer from benign or metastases, but 3D analysis was superior to 2D for the discrimination of benign nodules from metastases.

Fernandes and Kanehisa [49] used interior surfaces generated by voxel’s joint attributes and distribution for the classification of benign and malignant nodules. The volume was first pre-processed for surface extraction. Then, a 3D Alpha Shapes algorithm was used to reconstruct the surface and internal structures of the nodule. Shape distributions were measured by calculating five shape functions. Lastly, 163 features were selected to train a SVM. The dataset had 754 nodules (368 benign and 386 malignant) from LIDC-IDRI. The best performance was the sensitivity, specificity, and accuracy of 87.94%, 94.32%, and 91.05%, respectively. 

3D texture and margin sharpness features were used to classify small lung nodules with diameters between 3 and 10 mm [78]. 3D margin sharpness was implemented by computing the difference of intensity between lesions and its surroundings, and the sharpness of the intensity transition across the lesion boundary. The Genetic Algorithm was applied for feature selection. The classification model was evaluated on 274 small nodules. Three classifiers were compared (KNN, MLP, and Random Forest) and the Multilayer Perceptron (MLP) classifier achieved the best AUC of 0.82.

In Reference [50], regions containing nodules and surrounding parenchymas were labeled as nodule or parenchyma. Furthermore, 300 features including intensity, shape, border, and texture were employed. A 3D Laws’ Texture Energy Measures was applied to quantify the feature. Ray-casting and rubber-band straightening were used to remove border irregularity and histogram features were represented for density of the nodule and parenchyma. The algorithm was evaluated on 50 solitary nodule cases (22 malignant, 28 benign) from NIH. AUC of 0.935 and accuracy of 92% were obtained with 47 features. A similar method was proposed in Reference [79], in which mean and variance of border irregularity were also included. The system was validated with 27 ROIs (10 benign and 17 malignant) and obtained an accuracy of 92.6%.

Ciompi and Jacobs [97] proposed a novel bag-of-frequencies descriptor for lung nodules. Based on intensity morphological features and clustering, the label histogram was obtained. The method was validated on NELSON and DLCST. Han and Zhang [80] employed 3D Haralick texture features and SVM for classification. A total of 905 nodules from LIDC-IDRI were used and the AUC of 0.9441 was reached.

In References [51,52], 3D nodule segmentation was performed. Spherical harmonics (SHs) were computed for 3D surface representation. KNN with the number of SHs was employed for classification of benign and malignant nodules. In addition, 109 nodules from LIDC were used for the experiments. The accuracy of 94.4% was achieved in Reference [51]. In Reference [52], 327 nodules were used and the accuracy was 93.6%. El-Baz and Gimel’farb [98] used a rotation invariant second-order Markov-Gibbs random field to model the distribution of intensities for nodule representation. The approach used 109 nodules from LIDC and achieved the accuracy of 96.3%.

Namin and Moghaddam [53] presented volumetric shape index (SI) and fuzzy KNN-based method for lung nodule classification. Pre-processing was implemented for data normalization, ROI segmentation, noise reduction, and enhancement. The SI was calculated by the Hessian matrix. The fuzzy KNN classifier with multiple types of features (sphericity, gray level, and shape) from 3-D nodule reconstruction was used for classification. The system was evaluated on 134 nodules from LIDC and it achieved a sensitivity of 88%. 

In Reference [81], region growing was employed for segmentation. The nodule was represented by the Simposon’s Index, combined with 3D geometrical features (spherical disproportion, spherical density, and sphericity). One-Class SVM was trained. Thirty-nine nodules (30 benign and 9 malignant) from a private dataset were used. The algorithm obtained an accuracy, specificity, and sensitivity of 100%, 100%, and 100%, respectively.

In Reference [82], 3D active contour optimized by a simplex optimization method was applied for segmentation. Then, morphological and gray-level features were extracted from surrounding voxels with the rubber band straightening transform. LDC was trained for feature selection and classification. The method was evaluated on 23 nodules from the LIDC and achieved AUC of 0.83 ± 0.04. Based on Reference [82], Way and Sahiner [54] designed new features, which included surface smoothness, shape irregularity, and demographic features. A 2-loop leave-one-out resampling was designed as a classifier. Furthermore, 256 lung nodules (132 benign and 124 malignant) were tested. The AUC was improved to 0.863 ± 0.022. 

3D morphological and gray-level features were used to locate nodule in Reference [88], and LDA was employed for classification. The algorithm was validated on 470 nodules (69 malignant and 401 benign) with diameters between 3 to 30 mm and it gained an AUC of 0.79.

In Reference [55], 3D nodule ROIs were first segmented. The vascularity, shape, and density distributions were calculated to train ANNs with back-propagation for distinguishing benign and malignant nodules. The algorithm was evaluated on 48 cases (24 benign and 24 malignant) and gained the AUC of 0.89.

In Reference [56], the surrounding and internal structures of nodules were represented by the principal axes, compactness, and density value distribution pattern. 3D curvature indexes were used to construct the similarity measurement. The Mahalanobis distance was adopted as the difference measure between the representation nodules of the indeterminate cases and the retrieved lesions. Additionally, 107 cases (70 malignant and 37 benign) were used. The algorithm achieved a sensitivity of 91.4%, specificity of 51.4%, and accuracy of 77.6%. Topological and histogram features based on 3D curvature indexes were proposed in Reference [57]. K-means and LDA were used as a classifier. In addition, 210 nodules were used and the AUC of 0.97 was achieved. In Reference [58], nodules were clustered into two classes based on mean CT density. The LDA classifier was trained for each class based on internal structure features and 3D curvatures. A total of 248 nodules were used. 

Wyckoff and McNitt-Gra [59] combined contrast enhancement, 3D shape, and texture features with a linear classifier. A semi-automated contouring procedure was used for segmentation. Twenty-one cases were used for evaluation and the method achieved an accuracy of 95.2% by resubstituting and 81% by jackknifing.

In Reference [124], 2-pathway (accept a 3D volume of 50 pixel × 50 pixel × 5 slice and 100 pixel × 100 pixel × 10 slice, respectively) CNNs, including basic 3D CNNs, 3D multi-output CNNs, 3D DenseNet, and 3D DenseNet with multi-outputs were proposed. In addition, 686 nodules (54% malignant and 46% benign, the median malignancy value was computed) from LIDC-IDRI and 147 CT scans (63% malignant and 37% benign) from their own private database were used for evaluation. 3D multi-output DenseNet achieved the best performance with accuracy of 90.40% and AUC of 0.9548.

Xie and Xia [60] proposed a multi-view knowledge-based collaborative (MV-KBC) for classification of benign and malignant. The 3D nodule was decomposed into nine views. A KBC sub-model constructed by ResNet-50 networks was used for characterizing appearance, voxel, and shape with three types of image patches for each view as input. The results were obtained by weighting the output of nine sub models. The model was evaluated on LIDC-IDRI and achieved an AUC and accuracy of 0.9570% and 91.6%, respectively.

In Reference [125], radiological quantitative features were extracted and 3D nodule volumes were used as CNNs input. More than 1000 nodules from LIDC-IDRI were selected. This method got an AUC of 0.97 and high accuracy for nodule classification. When combined with quantitative image features (QIF), the result reached an AUC of 0.99.

In Reference [126], a 3D Faster R-CNN with UNet was employed to generate nodule candidates. Then the 3D dual path network was used to extract deep features. The gradient boosting machine with nodule size, raw pixels, and deep features was applied for classification. The algorithm was evaluated on LIDC-IDRI and obtained an accuracy of 90.44%. 

Kang and Liu [127] studied nodule classification based on the combination of different 3D CNNs and multi-view strategy. The 3D CNNs with chain and directed acyclic graph (DAG) were constructed by 3D Inception and 3D Inception-ResNet. Multi-view architecture was used to improve the performance. They used the 1 × 1 × 1 convolutional layer and the average pooling to replace the fully connected layer. The method was validated with 96 cases from LIDC-IDRI and it achieved an error rate of 4.59% for the binary classification and 7.70% for the ternary classification. Dou and Chen [105] combined multi-level context into three 3D CNNs models with various receptive fields. The approach was evaluated on LUNA16 and ELCAP.

A 3D CNNs method was proposed in Reference [128]. The 3D CNNs had four sets of 3D convolution-ReLU-pooling (2 × 2 max-pooling) layers, followed by two fully connected layers and a softmax layer. 1882 nodules from LIDC-IDRI with discarding score of 3 were utilized. The patch size of the input image was 64 pixel × 64 pixel × 5 slice for training. For performance evaluation, three strategies were used: 2D slice-level, 2D nodule-level, and full 3D nodule-level. They obtained classification accuracies of 86.7%, 87.3%, and 87.4%, respectively. The results showed that nodule-level 2D CNNs model can capture the z-direction well and nodule-level 3D CNNs can further integrate nodule-level and context features, which may be slightly better than the other two methods with limited situations.

Multi-View Convolutional Neural Networks were used in Reference [129]. Nine 2D views were extracted for each 3D candidate and the final results were computed by fusing CNNs outputs. This method was evaluated on LIDC-IDRI.

Shen and Zhou [110] proposed a Multi-scale CNNs model to distinguish benign and malignant nodules. This method first captured the wide range of nodule variability by extracting multiple patches. The patches were then fed to three CNNs to generate discriminative features. Random Forest and SVM classifiers were trained. In addition, 880 benign and 495 malignant nodules were collected from LIDC-IDRI for evaluation. Three scales including 32 pixel × 32 pixel × 32 pixel, 64 pixel × 64 pixel × 64 pixel, and 96 pixel × 96 pixel × 96 pixel were used as inputs. The method achieved 86.84% accuracy.

### 6.5. Other Methods

Other methods include clustering, semi-supervision, and hybrid features. They appeared in References [132,133].

Zhang and Song [132] developed a method for four-type lung nodule classification based on a semi-supervised method. A bipartite graph was constructed to present the direct similarities between training dataset (labeled images) and testing dataset (unlabeled images). Then, ranking score calculation was used to compute the possibility of each type that a testing image belongs to. The method was validated on 379 cases from ELCAP.

Antonelli and Cococcioni [133] proposed a set of classifiers and combiners based on decision templates technique. They obtained a sensitivity of 95% and specificity of 91.33% evaluated on 29 malignant and 37 benign nodules.

### 6.6. Summarization

Most of the works using traditional machine learning methods focuses on feature extraction, and then provides these features to the classifier for the classification task. The mainstream advanced classifiers include SVM [135], KNN [136], RF [137], and so on. Regarding the different deep learning methods employed in the selected works, part of them use neural networks [138] to extract features, and then input these features into classifiers. Another part of the works uses a combination of CNNs and softmax classifiers to achieve end-to-end classification. 3D image-based features can be obtained by traditional machine learning features or deep features by 3D CNNs. 

Table 4 and Table 5 summarize the methods and the results of selected works. For each of them, the tables provide information such as author, year of publication, datasets, and performance. Regarding the performance, indicators such as sensitivity, specificity, false positives, accuracy, and AUC are used.

## 7. Discussion

### 7.1. Discussion on Classification Performance

**Figure 8:**
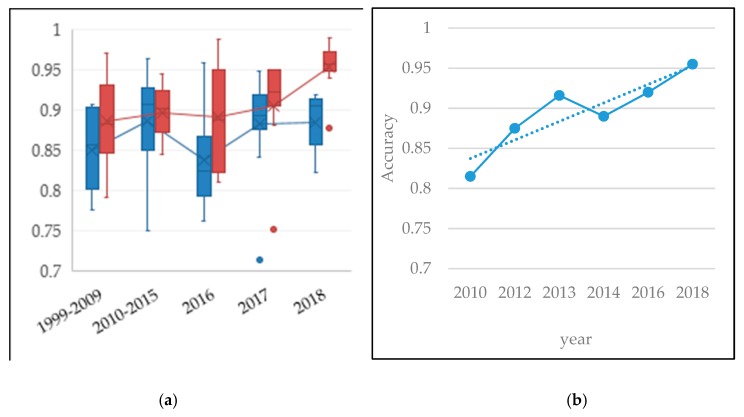
Trend of performance for selected papers: (**a**) the performance of two-type classification. The blue and red boxes indicate the accuracy and AUC, respectively. Each box indicates the worst, best, and median performance. (**b**) The performance of four-type classification.

It is clearly seen that lung nodule image classification is always a crucial research field and many research studies are published every year. Figure 8 depicts the changes in the performances of these published algorithms over the past few years, and the overall performance is continually increasing. Some methods have achieved an accuracy of more than 90%. However, there are still many problems to be discussed: (i) different datasets are used for evaluation. The performance of various methods cannot be treated equally and (ii) most performance indexes are obtained on relatively small datasets (smaller than 1000 cases). It is imperfect on statistical significance. (iii) Some methods are performed on the private dataset. It is hard to re-implement the algorithm and is not conducive to further study.

### 7.2. Discussion on the Adopted Method

Lung nodule image classification is essentially a problem of classification in the machine learning field, in which feature extraction and expression are leading roles compared to classifier construction. Therefore, feature engineering is the research focus. 

**Figure 9:**
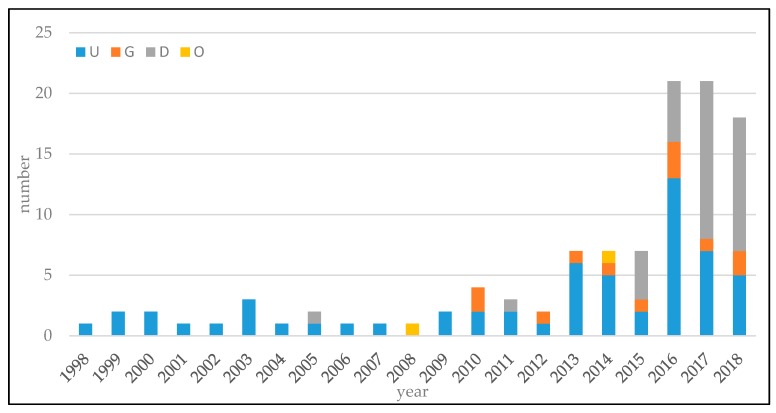
Trends of the technology used in this field. For the convenience of observation, the 3D feature methods merge into others.

Figure 9 shows the trends of technologies in this field. The works before 2010 mainly used user-defined feature-based methods. Deep feature-based methods are dominant on the latest research. Apparently, technology improvement of machine learning and computer vision fields led progress on lung nodule image classification. 

The advantage of user-defined features are that they are relatively intuitive and have good interpretability. Liu and Hou [18] achieved an accuracy of 92.3% with statistical and intensity features. Wei and Cao [74] showed an accuracy of 91.8% and AUC of 0.986 with texture and semantic features. Sasidhar and Geetha [75] had 92% accuracy using only texture features. Surrounding regions of nodules are incorporated to take more context information in some studies and they result in better classification than using nodule regions alone [134]. Some researchers also used patient history, gender, age, and other clinical features to obtain better decisions. However, these methods rely on professional understanding and analysis of the nodule image, which may have subjectivity. In addition, these methods lack uniformity, standardization, and universality. Nodule features extracted by hand crafted factors rely on the knowledge of experts in a specific field. It originates from simple visual intuition and it usually contains some strict assumptions. These assumptions are diverse, and it may not be useful since it is summarized and designed with insufficient medical image samples. 

Generic features extract the mid-level features of the lung nodule image. These methods have good mathematical expression and rigor and more computer scientists and engineers are devoted to this problem. These methods also have some limitations since they are not flexible enough to capture more complex patterns. Because nodule features extracted by generic methods are irrelevant to the task or objective and generic feature evaluation criteria are independent of specific application, the classification accuracy of selected features is usually lower than other methods. Most of these methods are combined with user-defined features and the performance can be improved. 

Deep features extract the high-level features of the lung nodule image. They have strong representative ability with the aid of massive training data and complex structure. Deep learning theory shows that it is advantageous compared with traditional machine learning methods. This is the way that visual cortices of mammalian and human work. Da Silva and da Silva Neto [113] used 3243 nodules and showed an accuracy of 94.78%. Liu and Kang [114] achieved an error rate of 5.41% for classification of benign and malignant. Dey and Lu [124] achieved an accuracy of 90.40%. The deep features can be combined with traditional features to obtain better representation of lung nodules. For example, Yuan and Liu [96] achieved the accuracy of 92.3% for four-type classification using statistical, geometric, histogram, and deep features. Xie and Xia [42] used shape, voxel values, appearance, and deep features and had an accuracy of 93.40%. The multi-scale and multi-view networks are applied several times [18,60,96,110,113,114,127,129]. Deep neural networks have a theoretical guarantee that they are much better than shallow layer models [140]. Features are extracted and represented from lower to higher layer. More complex and appropriate componential feature structures can be represented and this brings exponential advantages. Especially for classification, deep learning transforms the representation into a higher and more abstract level. Using this composition, complex functions can be learned. Deep layers of feature representation focuses on important and discriminate information and ignores the irrelevant variations [141]. 

However, there are some limitations in the field of medical images in deep learning. One problem to be concerned is the overfitting due to a lack of high quality labeled training data. Moreover, deep learning algorithms are generally black-box processes. The interpretability of these methods is weak and it is not easy to integrate expert opinions. Meanwhile, how to balance the model scale with training speed and training accuracy is also a problem. They require intensive computational training and more expertise for tuning (i.e., model architecture and hyper-parameters).

Moreover, all traditional features, including user-defined and generic, are extracted independently. SVM is one of the most commonly used classifiers. These features or their pooled variation (bag of visual words, fisher vector) are trained with an SVM classifier. The main disadvantage is that there is no scheme to improve local or global nodule feature extraction and representation. Essentially, feature extraction and classification are disconnected from each other. Nodule features extracted with DCNNs are data driven and it provides an end-to-end mechanism. The loss can be back propagated to previous layers to improve feature extraction. Hence, the hierarchical relationships between features can be discovered automatically without laborious handcrafting of features. The lower layer of DCNNs extract basic features of edges, lines, and points. Yet, these basic features combined layer-by-layer, and important organ part or the whole organ can be better represented with small neurons of a higher layer. DCNNs can also preserve local feature relations and perform dimension reduction by parameter sharing. Fortunately, the era of medical big data boosts deep learning.

3D feature methods become more and more popular. They have better scope, and capture stereoscopic and all aspects information that can help feature representation. These methods can be adopted to user-defined features, generic features, and deep features. However, the algorithms based on 3D are time-consuming and not much better than the algorithms based on 2D. The efficiency of these methods has always been a problem and need to be solved urgently. The collection and organization of 3D datasets are also one of the reasons that restrict its development. Some existing deep models use the 2D CNNs or multi-view 2D CNNs to simulate the volume of 3D images to complete the classification of lung nodules. For example, Xie and Xia [60] decomposed 3D nodules into 9 views.

### 7.3. Discussion on Benchmark Datasets

This paper makes a comprehensive study on lung nodule CT image classification. Model learning and parameter optimization are key roles, and, therefore, lung nodule image datasets become particularly important for its functions regarding providing training data and benchmarks. Although there are already some datasets, fatal problems still exist.

In addition, datasets are insufficient and the largest one only contains thousands of data, which is smaller than dataset in other fields. This may be due to special restrictions of medical, moral, and law, but it weakens the technique improvement. Data augmentation and pre-processing are common solutions.

For the covered works, about half works used LIDC-IDRI and ELCAP datasets. Standards are varied in different works. For example, research studies on LIDC-IDRI define malignant and benign nodules, according to diverse labels, and the number of samples is imbalanced in different categories. Type tags of W, V, J, and P are not given in ELCAP, and different researchers often label it. Therefore, the requirement of standard and structured annotations for relevant images remains challenging.

### 7.4. Proposals for Future Research

Although the problem of lung nodule CT image classification has made great progress, there are still studies to be studied.

(i) Develop unified and open platform. Datasets can be shared and all researchers make studies under the same standard.
(ii) Study together with lung nodule detection or other tasks. The accurate diagnosis requires comprehensive information. Future research studies should not only be based on local regions for classification, but also on the anatomical location of regions.
(iii) Deal with noise and uncertain annotations. For example, malignant levels of nodules given in LIDC-IDRI did not reach a consensus. The number of uncertain samples is larger than the number of certain samples. Researchers should make effective use of these uncertain data to improve classification.
(iv) Combine knowledge in the field of computer vision and data analysis. With the development of computer vision, it is important to relate these advanced algorithms with general medical image analysis.
(v) Focus on research studies of transfer learning and unsupervised learning. Try to conduct deep network training by bypassing the requirement of large datasets.
(vi) Fuse the guidance of professional doctors with deep feature. The interpretability of the classification model requires greater attention. It can provide in-depth understanding of disease for radiologists, which might be the ultimate objective.
(vii) Mobile platform application. Design high-speed and automated method to decrease model complexity, training cost, and prediction time.

## 8. Conclusions

This paper makes an appraisal of lung nodule classification algorithms for CT images. Published works are first collected, and then problem statements and dataset description are performed. All the selected works are careful reviewed. These works are grouped into several kinds on the basis of lung nodule feature representation. Discussions are given for the performance, the adopted method, and the benchmark dataset. The proposals for future research are also proposed. 

It can be observed from this work that deep learning-based methods are dominant with better performance. A 3D feature-based method can provide more information to make lung nodule representation more comprehensive. We suggest that, in addition to design stronger models and algorithms, a close relationship between researchers and clinicians is needed for better understanding and interpretation.

## Figures and Tables

**Table 1:** Description from LIDC-IDRI.

Case	Nodule ID	X Loc.	Y Loc.	Z Loc.	Contour of the Nodule	Malignancy
0007	Nodule 001	194	290	37	((189,280), (188,281), …, (190,281), (189,280))	5
0007	IL057_159747	293	266	30	((289,280), (290,279), …, (288,279), (289,280))	5
0092	Nodule 004	179	282	12		
0092	Nodule 005	361	333	108		

**Table 2:** Format of lung nodule position.

Scan	Type	X	Y	Slice
W0001	Nodule	98	218	54
W0001	Nodule	54	224	170
W0003	Nodule	158	356	80
W0005	Nodule	120	247	66
W0006	Nodule	109	258	129
W0007	Nodule	70	224	111

**Table 3:** Format of lung nodule position.

Database	Sample Number	Classification
Shanghai Zhongshan hospital database (ZSDB)	CT images from 350 patients	MIA, AAH, AIS, IA
SPIE-AAPM Lung CT Challenge [23,24]	22,489 CT images from 70 series	malignant and benign
General Hospital of Guangzhou Military Command (GHGMC) dataset	180 benign and 120 malignant lung nodules	malignant and benign
NSCLC-Radiomics database [25,26]	13,482 CT images from 89 patients	malignant and benign
Lung Nodule Analysis challenge 2016 (LUNA16) [27]	888 CT scans	subset of LIDC-IDRI
Danish Lung Nodule Screening Trial (DLCST) [28]	CT images from 4104 participants	Nodule and non-nodule

MIA: Minimally Invasive Adenocarcinoma, AAH: Atypical Adenomatous Hyperplasia, AIS: Adenocarcinoma In Situ, and IA: Invasive Adenocarcinoma.

**Table 4:** Main techniques and comparative analysis of the two-type classification. U: user-defined features, G: Generic features, D: deep features, 3D: 3D features, O: other methods. Acc: accuracy, Sen: sensitivity, Spe: specificity.

Authors	Year	Database	Features	Classifier	Performance
Xie et al. [45]	2018	LIDC-IDRI	U, D	ANNs	AUC:0.9665, 0.9445 and 0.8124
Wei et al. [61]	2018	LIDC-IDRI	U	Spectral clustering	Error rate: 10.9%, 17.5%
Wei et al. [74]	2018	LIDC-IDRI	G	CBIR	AUC: 0.986, Acc: 91.8%
Dey et al. [124]	2018	LIDC-IDRI, Private	D, 3D	CNNs	Acc: 90.4%, AUC: 0.9548
Xie et al. [60]	2018	LIDC-IDRI	U, D, 3D	CNNs	AUC: 0.9570, Acc: 91.6%
Chen et al. [37]	2018	72 patients, 75 nodules	U	SVM	Acc: 84%, Sen: 92.85% Spe: 72.73%
Gong et al. [46]	2018	Private	U, 3D	SVM, LDA, Naïve Bayes	AUC: 0.94, 0.90, 0.99
Zhao et al. [108]	2018	LIDC-IDRI	D	CNNs	Acc: 82.2%, AUC: 0.877
Li et al. [73]	2018	LIDC-IDRI, private	G	RF	Sen: 92%, AUC: 0.95
Causey et al. [125]	2018	LIDC-IDRI	U, D,3D	RF	AUC: 0.99
Zhu et al. [126]	2018	LIDC-IDRI, LUNA16	D, 3D	CNNs, GBM	Acc: 90.44%
Tajbakhsh et al. [121]	2017	415 cases, 489 nodules	D	MTANNs, CNNs	AUC: 0.8806 and 0.7755
Shen et al. [123]	2017	LIDC-IDRI	3D, D	CNNs	Acc: 87.14%, AUC: 0.93
Hancock et al. [93]	2017	LIDC-IDRI	U	Linear classifier	Acc: 88.08%, AUC: 0.949
Xie et al. [42]	2017	LIDC-IDRI	U, D	CNNs	Acc: 93.40%
Le et al. [86]	2017	ZSDB, LIDC-IDRI	U	RF	AUC: 0.9144 and 0.8234 Acc: 89.20% and 82.92%
Kang et al. [127]	2017	LIDC-IDRI	D, 3D	CNNs	Error rate: 4.59%
Wei et al. [87]	2017	LIDC-IDRI	U	CBIR	AUC: 0.751, Acc: 71.3%
Jin et al. [111]	2017	LIDC	D	CDBNs	Acc: 92.83%
Song et al. [112]	2017	LIDC-IDRI	D	CNNs, DNN, SAEs	Acc: 84.15%, Sen: 83.96% Spe: 84.32%
Silva et al. [113]	2017	LIDC-IDRI	D	CNNs	Sen: 94.66%, Spe: 95.14% Acc: 94.78%, AUC: 0.949
Nibali et al. [103]	2017	LIDC-IDRI	D	CNNs	Acc: 89.90%
Xu et al. [115]	2017	LIDC-IDRI	D	SVM	Acc: 89%, AUC: 0.95
Jiang et al. [29]	2017	LIDC-IDRI	U	SVM, RF, KNN, CNNs	Acc: 77.29%, 80.07%, 84.21%; AUC: 0.913
Paing et al. [130]	2017	TCIA [139]	U, 3D	SVM	Acc: 90.9%
Shen et al. [123]	2017	LIDC-IDRI	3D, D	CNNs	Acc: 87.14%, AUC: 0.93
Dhara et al. [47]	2016	LIDC-IDRI	U, 3D	SVM	AUC: 0.9505, 0.8822 and 0.8848
Yan et al. [128]	2016	LIDC-IDRI	D, 3D	CNNs	Acc: 86.7%, 87.3%, and 87.4%
Sasidhar et al. [75]	2016	LIDC-IDRI	U, G	SVM	Acc: 92%
Htwe et al. [67]	2016	LIDC-IDRI, SPIE-AAPM	U	Fuzzy system	Sen: 87%, Acc: 78%
Dhara et al. [34]	2016	LIDC-IDRI	U	SVM	AUC: 0.9465
Gierada et al. [48]	2016	94 patients, 96 nodules	U, 3D	Regression analysis	AUC: from 0.79 to 0.83
Sergeeva et al. [62]	2016	LIDC-IDRI	U	KNN	Acc: 81.3%
Fernandes et al. [49]	2016	754 nodules	U, 3D	SVM	Sen: 87.94%, Spe: 94.32% Acc: 91.05%
Shewaye et al. [94]	2016	LIDC-IDRI, Private	U, G	SVM, KNN, RF, Logistic Regression, AdaBoost	Acc: 82% of malignant and 93% of benign
Rendon-Gonzalez et al. [35]	2016	SPIE-AAPM	U	SVM	Acc: 78.08%, Sen: 84.93% Spe: 80.92%
Kim et al. [92]	2016	Private	U, D	SVM	Acc: 95.5%, Sen: 94.4% AUC: 0.987
Ma et al. [30]	2016	TCIA	U	RF	Acc: 82.7%
Liu et al. [114]	2016	LIDC-IDRI	D	CNNs	Error rate: 5.41%
Felix et al. [78]	2016	274 nodules	U, 3D	MLP, KNN, RF	AUC: 0.82
Sun et al. [120]	2016	LIDC-IDRI	D	CNNs,DBNs SDAE	Acc: 79.76%, 81.19% and 79.29%
Wang et al. [36]	2016	LIDC-IDRI	U	SVM	Acc: 76.1%
Huang et al. [131]	2016	100 series	U	Logistic regression	Acc: 79%; AUC: 0.81
Song et al. [31]	2016	LIDC	U		Acc: 83.4%
Xie et al. [44]	2016	LIDC-IDRI	U, D	CNNs	Acc: 86.79%;
Aggarwal et al. [39]	2015	Private	U	SVM	Acc: 82.32%
Narayanan et al. [69]	2015	LIDC	U	ANNs	Acc: 92.2%, FP: 0.9%
Dilger et al. [50]	2015	50 nodules	U, G, 3D	ANNs	AUC: 0.935, Acc: 92%
Hua et al. [118]	2015	LIDC	D	CNNs	Sen: 73.4% and 73.3% Spe: 82.2% and 78.7%
Kumar et al. [119]	2015	LIDC-IDRI	D	binary decision tree	Acc: 75.01%, Sen: 83.35%
Shen et al. [110]	2015	LIDC-IDRI	3D, D	SVM, RF	Acc: 86.84%
Tartar et al. [91]	2014	Private	U	AdaBoost, Bagging, RSS	Sen: 94.7%, 90.0%, 77.8% Acc: 89.5%
Dandil et al. [66]	2014	47 patients, 128 nodules	U	ANNs	Acc: 90.63%, Sen: 92.30% Spe: 89.47%
Huang et al. [100]	2013	107 images	U	SVM	Acc: 83.11%, AUC: 0.8437
Dilger et al. [79]	2013	27 nodules	U, 3D	NN	Acc: 92.6%
Han et al. [80]	2013	LIDC-IDRI	U, 3D	SVM	AUC: 0.9441
Lin et al. [101]	2013	107 scans	U	SVM	AUC: 0.9019, Acc: 88.82% Sen: 93.92%, Spe: 82.90%
Nascimento et al. [63]	2012	LIDC	U	SVM	Sen: 85.64, Spe: 97.89% Acc: 92.78%
El-Baz et al. [51]	2011	LIDC	U, 3D	KNN	Acc: 94.4%
Chen et al. [77]	2011	47 nodules	D	BPNN, RBPNN, LVQNN	Acc: 78.7%
El-Baz et al. [52]	2011	LIDC	U, 3D	KNN	Acc: 93.6%
Namin et al. [53]	2010	LIDC	U, 3D	KNN	Sen: 88%
El-Baz et al. [98]	2010	LIDC	U, 3D	Bayes	Acc: 96.3%
Silva et al. [81]	2009	Private	U, 3D	SVM	Acc: 100%, Spe: 100% Sen: 100%
Way et al. [54]	2009	Private	U, 3D		AUC: 0.863
Antonelli et al. [133]	2008	66 nodules	O		Sen: 95%, Spe: 91.33%
Way et al. [82]	2006	LIDC	U, 3D	LDA	AUC: 0.83
Suzuki et al. [122]	2005	489 nodules	D	ANNs	AUC: 0.882
Armato et al. [88]	2003	393 scans, 470 nodules	U, 3D	k-means	AUC: 0.79
Lo et al. [55]	2003	48 cases	U, 3D	ANNs	AUC: 0.89
Kawata et al. [56]	2003	107 cases	U, 3D		Sen: 91.4%, Spe: 51.4% Acc: 77.6%
Kawata et al. [58]	2001	248 nodules	U, 3D	k-means, LDA	AUC: 0.97
Kawata et al. [57]	2000	210 nodules	U, 3D	k-means, LDA	AUC: 0.97
Wyckoff et al. [59]	2000	21 cases	3D, U		Acc: 81%
McNitt et al. [33]	1999	31 cases	U	LDA	Acc: 90.3%

**Table 5:** Main techniques and comparative analysis of the selected four-type classification. U: User-defined features. G: Generic features. D: deep features. 3D: 3D features. O: other methods. Acc: accuracy. Sen: sensitivity. Spe: specificity.

Author	Year	Database	Features	Classifier	Performance
Liu et al. [18]	2018	LIDC-IDRI, ELCAP	U, D, 3D	CNNs	Acc: 92.3% and 90.3%
Yuan et al. [96]	2018		U, G, D, 3D	SVM	Acc: 93.1% and 93.9%
Mao et al. [95]	2018	ELCAP	U, D	Softmax	Acc: 95.5%
Mao et al. [99]	2016	ELCAP	U	SVM, clustering	Acc: over 90%
Mao et al. [107]	2016	ELCAP	G	Ensemble classifier	Acc: 92%
Zhang et al. [70]	2014	ELCAP	U, G	SVM, pLSA	Acc: 89%
Zhang et al. [132]	2014	ELCAP	O		Acc: about 88%
Zhang et al. [102]	2013	ELCAP	U	SVM	Acc: 82.5%
Zhang et al. [17]	2013	ELCAP	G	CPMw	Precision: 0.916
Song et al. [71]	2012	ELCAP	U, G	SVM	Acc: about 87.5%
Farag et al. [76]	2010	ELCAP	G	LDA	Acc: 81.5%
Farag et al. [72]	2010	ELCAP	G	LDA	Acc: 78.23%
